# The Relation of Certain Urinary Findings to Prognosis on Pulmonary Tuberculosis

**Published:** 1915-11

**Authors:** J. C. Cummings

**Affiliations:** Sacramento, Cal.


**The Relation of Certain Urinary Findings to Prognosis on Pulmonary Tuberculosis.**. By J. C. Cummings, M. D., Sacramento, Cal. California State Journal of Medicine, Oct. 1915. In making the so-called urochromogen test the technic of Weiss was used. This requires a small amount of fresh urine diluted with three parts of water, the mixture is thoroughly shaken up and divided into two equal portions in test tubes. To one of these portions three drops of potassium permanganate solution is added, the other tube being used as a control. Many specimens were turbid and of various shades of amber, but the turbidity and original color seemed to have little effect upon the reaction. I recognized as a positive only a distinctly canary yellow which disappeared rather slowly upon standing. A diazo was done upon each specimen.

In 100 successive admissions of patients suffering from pulmonary tuberculosis only 14 could be given a favorable clinical prognosis. In no case considered as "favorable" from a clinical standpoint did the so-called urochromogen reaction appear in the urine. In seven of 14 cases (50%) clinically considered "favorable" a positive diazo reaction was obtained. In 39 of 86 (45%) clinically unfavorable cases the urochromogen test was positive while in 53 (61%) the diazo test was positive. Seventeen of 100 patients died in six months. Of these 17, 11 (65%) showed a positive urochromogen, and 13 (76%) a positive diazo while 10 (59%) showed both. Of 17 patients showing a positive urochromogen test with a negative diazo (a combination considered especially ill-omened by Weiss) none have died to date. Of 39 patients having a negative urochromogen and a positive diazo, five have died. The results are more compactly shown in the accompanying table.

Conclusions.

(1) The urochromogen and diazo reactions appear in the urine of a majority of patients in a late stage of pulmonary tuberculosis.

(2) They do not appear until long after a correctly unfavorable prognosis is possible by a careful clinical examination.

References—1. Weiss. Journ. Am. Med. Ass'n., Nov. 22, 1913, p. 1943. 2. Heflebower. Am. Journ. Med. Sc., 1912, p. 221. 3. Schaffle. Journ. Am. Med. Ass'n., Oct. 10, 1914, p. 1294.

